# The Interactive Effects of Personal Resources on Teachers’ Work Engagement and Withdrawal Intentions: A Structural Equation Modeling Approach

**DOI:** 10.3390/ijerph17072170

**Published:** 2020-03-25

**Authors:** Sergio Mérida-López, Natalio Extremera, Nicolás Sánchez-Álvarez

**Affiliations:** 1 Faculty of Psychology, Department of Social Psychology, Social Work, Social Anthropology and East Asian Studies, University of Málaga, 29071 Málaga, Spain; nextremera@uma.es; 2 Faculty of Psychology, Department of Basic Psychology, University of Málaga, 29071 Málaga, Spain; nsa@uma.es

**Keywords:** emotional intelligence, work engagement, withdrawal intention, teacher self-efficacy, structural equation modeling

## Abstract

This research contributes to the current knowledge on teacher well-being by examining an integrated model with a personal resource (i.e., emotional intelligence) explaining teacher withdrawal intention through a mediator (i.e., work engagement) and considering the moderator effect of a second personal resource (i.e., teacher self-efficacy) in this relationship. Adopting a cross-sectional design, a total of 702 teachers (63.2% female) working at different educational levels took part in this study. The results showed that emotional intelligence and teacher self-efficacy were positively related to work engagement and negatively related to withdrawal intentions. Most importantly, the results demonstrated support for the hypothesized model—that is, teacher self-efficacy moderated the relationship between emotional intelligence and work engagement. Taken together, our findings highlight both emotional intelligence and teacher self-efficacy as positive individual resources for increased work engagement and reduced withdrawal intentions. This study has implications for the development of intervention programs aiming at increasing occupational well-being in educational settings.

## 1. Introduction

Despite the mounting body of research on teacher engagement [1], teacher well-being [2], and teacher commitment [3], issues of teacher shortages remain a concern for policymakers and practitioners worldwide. In recent decades, attrition rates have increased to the point that studies have reported that more teachers leave voluntarily in comparison to those who remain in the classroom until retirement [4]. Teacher attrition represents a major obstacle for stability within this occupational field, thereby having a deep impact on educational settings and society [5]. Hiring and training replacements is not only costly [6]; students must also deal with the detrimental effects of reduced teacher well-being and increased withdrawal intentions [7].

In light of the above-described findings, teacher attrition has emerged as an important worldwide issue for scholars [6,8] and educational administrations [9,10]. Although expressing the intention to withdraw does not entirely correspond to actual withdrawal, there is strong support for causality between behavior intentions and implementations, so measures of intention can accurately account for significant levels of actual behavior [11,12]. Withdrawal intention conveys feelings and decisions about a profession that are antecedents of initiation of turnover behaviors. Consistently, previous research has assessed withdrawal intention as a predictor of eventual turnover [11,13]. 

In recent years, researchers and practitioners have directed their attention to the individual characteristics predicting health, motivation, and well-being in organizational contexts [14]. Previous studies have put efforts into examining psychological resources as antecedents of teachers’ decisions about remaining in their positions, considering factors such as self-efficacy and emotional intelligence [15,16,17]. 

This study underlines the role of emotional intelligence and teacher self-efficacy to explain work-related well-being and withdrawal intention. On the one hand, Hong’s research [16] has provided insightful data on the individual differences between leavers and stayers regarding psychological resources. In this study, reduced self-efficacy was found to be a salient predictor of teacher attrition. Conversely, there is extensive evidence supporting that teachers’ beliefs in their capabilities to accomplish profession-related activities (e.g., using instructional strategies or establishing a classroom management system) are positively associated with their satisfaction and occupational commitment [3,17]. On the other hand, there are differences in teachers’ strategies to manage the negative emotions related to their work that may predict eventual attrition [16]. Indeed, the teachers’ abilities to effectively process affective information play a role in their levels of well-being, satisfaction, and commitment [18]. In sum, it has been argued that teachers’ personal resources may facilitate retention over the long term [15]. Given that these resources have been shown to be malleable through interventions [14,19], this approach may be a relevant way of improving teacher retention. 

**Figure 1:**
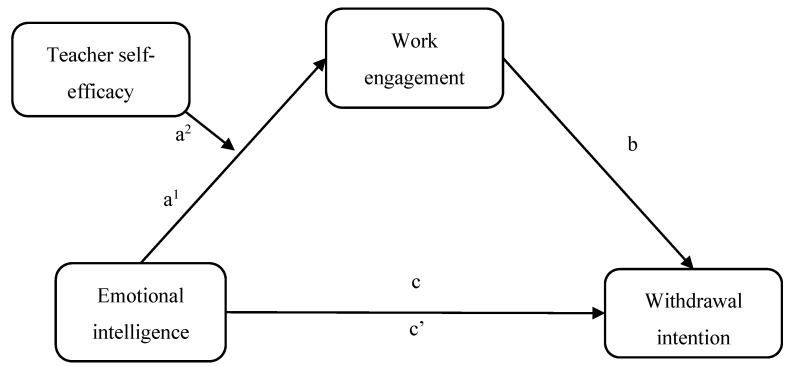
Representation of the proposed model. Note: a^1^ = direct effect of emotional intelligence; a^2^ = direct effect of teacher self-efficacy; b = total effect of work engagement; c = total effect of emotional intelligence; c’ = direct effect of emotional intelligence.

By means of the Job Demands-Resources and Emotional Intelligence theories [20,21], the primary goal of this study was to gain more insight into the main and interactive effects of emotional intelligence and self-efficacy as personal resources explaining teachers’ withdrawal intentions through work engagement (see Figure 1). In doing so, this work would contribute to the teacher education field aiming at promoting occupational well-being, commitment, and retention.

### 1.1. Emotional Intelligence and Teachers’ Withdrawal Intentions

One significant update from the original version of the Job Demands-Resource (JD-R) model is the integration of personal resources other than job resources [20]. Following the revised version of the JD-R model, personal resources are defined as “those aspects of the self that are generally linked to resilience and refer to the individuals’ sense of their ability to control and impact upon their environment successfully” [20,22]. Accordingly, personal resources influence work motivation as they help workers to deal with the adverse effects of job demands [23]. Examples of personal resources are optimism [22], psychological capital [24], or emotional intelligence [25,26]. Workers high in personal resources are more likely to accept setbacks and failures as normal instead of a lack of worthiness because they have a more resilient way to perceive and deal with daily job demands [27]. In this study, we focus on emotional intelligence (EI) because it has been demonstrated to have key implications for teachers’ work motivation and commitment [28,29].

Unlike trait models defining the EI construct with a broader perspective that understands it as the tendency of an individual to manage their emotions, the ability EI model conceptualizes EI as the ability to perceive, assimilate, understand, and manage emotions in oneself and others [30,31]. We follow the latter model because it has received extensive theoretical and empirical support [21]. The EI construct has become a topic of interest receiving a growing amount of attention in teaching and teacher education literatures [18]. Indeed, there is substantial evidence on the relevance of EI for achieving better functioning within organizational settings [32]. Several meta-analytic reviews have shown that EI is associated with health [33], well-being [34], work attitudes [28], and performance [35]. 

A recent review of the literature on emotions in the workplace has proposed a theoretical model in which workers’ abilities to deal with events and affectivity play a significant role in their withdrawal intentions [36]. Accordingly, findings from Hong’s study [16] support the need to develop a research agenda that sheds more light on the association between teachers’ differences in emotional skills and their decisions about leaving teaching. This examination seems mandatory in an occupational context such as teaching owing to the fact that teacher attrition is a major concern worldwide [8,9]. Considering recent meta-analytic evidence on the associations between EI and turnover intentions [28], it is plausible to expect low levels of EI to be linked with higher teachers’ withdrawal intentions. This assumption is in line with earlier research stating EI as a valuable individual resource for improving teaching effectiveness [18].

Regarding the potential mechanisms explaining how emotionally intelligent teachers are more committed to their occupations and, thus, less likely to leave their careers, current data has demonstrated that EI might facilitate social relationships at work and thus help workers to feel more satisfied [37]. There is evidence that shows EI as a relevant factor in relation to distal antecedents of teachers’ withdrawal intentions such as workplace social support [38] or classroom management [39]. Similarly, there is support for the relationship between EI and proximal antecedents of withdrawal such as teacher burnout [40] and teacher satisfaction [41]. Therefore, it is expectable that those teachers with higher EI report lower scores in withdrawal intention than their counterparts with lower EI. We propose the following:

**Hypothesis 1 (H1).**  *There is a negative relationship between EI and withdrawal intentions.*

### 1.2. The Mediating Role of Work Engagement in the Relationship between EI and Teachers’ Withdrawal Intentions

Work engagement is generally defined as a positive, fulfilling, work-related state of mind characterized by vigor, dedication, and absorption [42]. Work engagement has received a growing deal of attention in the teacher education literature as a pivotal aspect of work-related well-being, as it affects a wide variety of individual and organizational outcomes [43,44]. With regard to individual antecedents of work engagement, there is growing literature examining workers’ individual characteristics beyond workplace factors [27,44]. Studies conducted through the JD-R model lens agree on the predictive effects of personal resources such as psychological capital or optimism on work engagement [24,45]. These studies have shown that these resources help workers in dealing with work demands and ultimately allow them to feel more engaged with their work. This accords with the proposition of the JD-R model that personal resources directly influence well-being [20,23]. Nonetheless, there is need for empirical support regarding the underlying motivational mechanisms through which a personal resource such as EI would negatively relate to withdrawal intentions. 

There is growing evidence suggesting that EI would play a significant role as a personal resource within the JD-R model [25,46]. There are studies reporting evidence on the motivational effects of teachers’ abilities to deal with one’s own and others’ emotions [47]. Moreover, EI levels could account for the significant variance in workers’ levels of engagement regardless of their personality [48]. Studies showing positive associations between EI and work engagement can be found across different occupational settings including teaching [25,46,49]. Accordingly, one would expect teachers high in EI to report higher levels of work engagement than their low-EI counterparts. In sum, the evidence suggests emotionally intelligent teachers to be more skilled at shaping their mood so they can make the most of their motivational and cognitive resources to solve problems at work [36]. This accords with the importance of emotional abilities for promoting social relationships or dealing with work stress [21]. 

Workers perceiving their work as engaging might find it easier to get involved and to dedicate more time to their tasks. As a consequence of such efforts and involvement in their work, they would be less likely to leave their profession [44]. Prior research has reported work engagement to be negatively related to teachers’ withdrawal intentions [50]. Finally, work engagement has been proposed as a mechanism relating EI with reduced turnover intentions [51]. Based on prior research, we propose the following:

**Hypothesis 2 (H2).**  *Work engagement mediates the relationship between EI and withdrawal intention.*

### 1.3. The Moderating Role of Teacher Self-Efficacy in the Relationship between EI and Work Engagement

Teacher self-efficacy is defined as “a teacher’s beliefs in her or his capability to bring about student engagement and learning outcomes even when the students are challenging” [52]. Current knowledge supports the relevance of this individual resource, as high self-efficacious teachers report less stress [53] and burnout [54]. Moreover, teachers scoring higher in self-efficacy feel more committed to their careers [3], and they experience greater well-being [2] and satisfaction with their jobs [55]. According to the JD-R model, teachers perceiving themselves as self-efficacious are seen as more likely to exhibit a strong investment in their work, set higher goals, or exert more effort to research those goals [20,52]. Consequently, workers who have a high sense of efficacy are more likely to feel engaged at work than those with a low sense of efficacy. There is evidence of positive associations between teacher self-efficacy and work engagement [17,56,57]. 

Following a recent literature review of the implications of EI in organizational settings, there is need for work examining how EI interacts with other individual characteristics to predict workplace outcomes [32]. The above-described studies empirically supported the separate contributions of EI and self-efficacy as personal resources explaining health, motivation, and well-being in teaching. To date, however, no study has tested the interactive contribution of EI with another personal resource (i.e., teacher self-efficacy) in the prediction of work engagement. Prior research has found differences between teachers who stay or leave their career regarding their psychological resources and their responses to challenging situations at work [16]. Therefore, examining teacher self-efficacy as a potential moderator in the relationship between EI and work engagement and the potential benefits teachers may gain from fostering EI abilities must be addressed so that researchers, school counselors, and policy makers move forward with teacher-mentoring programs as effective ways to contribute to teacher retention.

Although prior research shows that both leavers and stayers report classroom management as a stressor, those who remain in a teaching career appear to implement different strategies to reduce their likelihood of burning out [16]. Therefore, it would be reasonable to similarly find between-person differences regarding the association between EI and work engagement with regard to teacher self-efficacy. Considering the situation-specific model of EI [21], it is expected that certain individual characteristics such as teacher self-efficacy might modulate the relationship between EI and work engagement. According to the predictive effects of self-efficacy on work engagement [17,45], it could be anticipated that teacher self-efficacy would strengthen the impact of EI on work engagement. In sum, based upon Côté’s model [21] and the available empirical research, we expect teacher self-efficacy to moderate the relationship between EI and work engagement. We propose the following:

**Hypothesis 3 (H3).**  *Teacher self-efficacy moderates the relationship between EI and work engagement. The higher the perceived teacher self-efficacy, the more positive the relationship between EI and work engagement.*

### 1.4. Rationale for This Study 

Previous studies have mostly investigated the unique predictive effects of teacher self-efficacy or emotional intelligence in several health, well-being, and performance outcomes [2,21]. However, the interactive effect of teachers’ personal resources on work engagement and associated effects on withdrawal intentions has not yet been empirically examined. Thus, our study was aimed at contributing to the teacher education field in both theoretical and practical ways. First, testing the interplay of emotional intelligence and self-efficacy as predictors of work engagement and withdrawal intentions would empirically help sustain the boundary conditions of the effects of emotional intelligence on teacher engagement considering a key driver of teachers’ occupational well-being such as self-efficacy [2]. This would expand our understanding of the motivational role individual resources play in teachers’ daily work lives. 

Second, we intend to make a practical contribution to the field of teacher education. From a primary prevention perspective, it is crucial for organizations to enhance workers’ personal resources not only as a profitable way of reducing future problems but also to promote occupational health and well-being [14,45]. This approach is also profitable in its own right, as it could lead to reducing costs associated with withdrawal [6,58]. Thus, our results could help both researchers and practitioners to design tailored interventions so work engagement is enhanced and subsequent retention is facilitated. 

## 2. Materials and Methods

### 2.1. Participants

The study sample was comprised of 702 educators (M_age_ = 44.38, SD = 8.94; 63.2% female) working in several educational centers in southern Spain (15% elementary teachers, 35.6% primary educators, 47.7% secondary teachers, and 1.7% did not report their teaching level). The participants had an average of 17 years of teaching experience. A total of 81.9% of the teachers worked in state schools, and 15.24% worked in private schools receiving public funds.

### 2.2. Measures

In addition to relevant sociodemographic data (i.e., age, gender, teaching level, and teaching experience), well-validated instruments were used to assess the study variables.

#### 2.2.1. Emotional Intelligence

Emotional intelligence was assessed with the Spanish version of the Wong and Law Emotional Intelligence Test (WLEIS) [59,60]. Participants were given a 7-point Likert scale ranging from (1) “Totally disagree” to (7) “Totally agree.” This instrument is comprised of 16 items and assesses four dimensions: self-emotion appraisal, other-emotion appraisal, use of emotion, and regulation of emotion. Sample items are: “I always encourage myself to try my best” and “I have a good sense of why I have certain feelings most of the time.” Since our interest was in the EI construct as a whole, we used the overall score as in prior research [60,61]. The Spanish version of the WLEIS has shown satisfactory validity and reliability [59]. 

#### 2.2.2. Teacher Self-Efficacy

Teacher self-efficacy was assessed using the short form of the Teachers’ Sense of Efficacy Scale-Short Form (TSES-SF) [52]. This instrument requires participants to assess the perceptions of their level of a range of abilities relevant to teaching. Respondents answered on a Likert-type scale (1 = “nothing” to 9 = “a great deal”). Through three 4-item subscales, the TSES-SF assesses self-efficacy beliefs regarding student engagement (e.g., “How much can you do to help students to value learning?”), classroom management (e.g., “How much can you do to control disruptive behavior in the classroom?”), and instructional strategies (e.g., “How much can you do to implement a variety of assessment strategies?”). However, the overall score was used as we were interested in the whole construct [41]. The Spanish adaptation has shown adequate psychometric properties with teacher samples [62].

#### 2.2.3. Work Engagement

Work engagement was measured with the Spanish version of the Utrecht Work Engagement Scale (UWES) [42]. Respondents were given 15 items and were requested to respond using a scale ranging from (0) “Never” to (6) “Always”. This instrument assesses three dimensions of work engagement: vigor, dedication, and absorption. However, in line with earlier studies, we used the overall score of work engagement, as we were more interested in the whole construct [46]. One example item is “My job inspires me”. A well-validated Spanish version of the scale was used [63].

#### 2.2.4. Withdrawal Intention

Withdrawal intention was assessed with the three-item occupational withdrawal intentions scale [64]. Participants were asked to use a 9-point scale ranging from (1) “Disagree strongly” to (9) “Agree strongly”. One example item is “I think about quitting the teaching profession”. The instrument was professionally translated from English into Spanish using the back-translation method. This Spanish version has shown adequate reliability [62].

### 2.3. Procedure

In line with prior research, a student-recruited sample was used to collect data from educators [65]. In short, participants were contacted with the help of university students who were instructed in the administration of surveys. Once the questionnaires were completed, the students returned them to the teaching staff for statistical processing. Participants were fully informed about the voluntary, individual, and confidential nature of the study and were aware that the topic of the research was the “personal and contextual factors relating to teacher motivation”. This study was carried out in accordance with the Declaration of Helsinki, and its ethical guidelines were approved by the Research Ethics Committee of the University of Málaga (66-2018-H).

### 2.4. Analytic Strategy

To verify the proposed hypotheses, the following analysis procedure was conducted. To test H1, bivariate Pearson correlation analysis was conducted to establish the significant associations between the variables. This analysis was a necessary condition to further test a mediator model. To test H2, four necessary conditions to establish mediation were examined [66]: 1) the independent and mediating variables must be significantly related; 2) the dependent and independent variables must exhibit a significant relationship; 3) the mediating and dependent variables must be significantly related; and 4) the relationship between the independent and dependent variable must be non-significant or weak when the mediating variable is included. After examining accomplishment of the necessary conditions for the mediation tests, H3 was tested by means of a Structural Equation Modeling (SEM) approach with AMOS 24. In this analysis, the relevant sociodemographic variables were controlled to avoid potential confounding effects. Finally, we used the PROCESS macro to determine the moderating effect of teacher self-efficacy on the relationship between EI and work engagement [67]. Specifically, the Johnson-Neyman technique was used to examine the significant values of the moderating variable effect [68].

Once the data screening was performed, the SPSS v23.0 statistical software (SPSS Inc., Chicago, IL, USA) was used to calculate the descriptive statistics, reliabilities, and correlations of the measured variables. To examine the factorial validity of the main study variables within our sample, a confirmatory factor analysis using structural equation modeling was conducted. AMOS 24 was used considering Maximum Likelihood Estimation. According to the recommendations of Hu and Bentler [69], goodness of fit was assessed with the χ^2^ index, the Comparative Fit Index (CFI), Tucker-Lewis Index (TLI), the Root Mean Square Error of Approximation (RMSEA), and the Standardized Root Mean Square Residual (SRMR). In general, TLI and CFI values of 0.95 or higher reflect a good fit. RMSEA values of less than 0.06 indicate an excellent fit, whereas values between 0.06 and 0.08 indicate an acceptable fit. Finally, SRMR values lower than 0.08 indicate an adequate fit. To determine the internal consistency of the instruments, we estimated the Cronbach’s α coefficient [70], Guttman’s λ [71], and McDonald’s ω [72]. 

## 3. Results

### 3.1. Factorial Validity and Reliability of the Measures

First, confirmatory factor analyses of the instruments used in this study were carried out. The results showed a factorial structure according to the original instruments as well as an appropriate fit (see Table 1). The factorial loads of each instrument were greater than 0.50, which indicates an appropriate contribution of each of the items to their corresponding factors [73]. The reliability results of the scales showed appropriate indexes between 0.90 and 0.94.

### 3.2. Descriptives

The descriptive statistics and correlation indexes are reported in Table 2. The results followed the expected pattern so that EI was positively related to work engagement and self-efficacy, and negatively associated with withdrawal intention. This latter association was in support of H1 and supported condition 2 for the mediation analysis such that the dependent (i.e., withdrawal intention) and independent (i.e., EI) variables are significant related. Likewise, EI and work engagement were significantly and positively related, supporting condition 1 such that the independent (i.e., EI) and mediating (i.e., work engagement) variables are significantly associated. Finally, a significant association was found between work engagement and withdrawal intention, supporting condition 3 such that the mediating (i.e., work engagement) and dependent (i.e., withdrawal intention) variables are significantly related.

### 3.3. Tests of Mediation

Regarding H2 on the mediator role of work engagement in the relationship between EI and withdrawal intention, we used SEM analyses to examine the association between EI and withdrawal intention through work engagement. The results indicated an excellent fit (*χ^2^* = 3.198, df = 4, CFI = 0.999, TLI = 0.999, RMSEA = 0.001, SRMR = 0.021). We used z-statistic significant testing to examine the indirect effect in line with the guidelines of Preacher et al. [74]. The mediation analyses of the direct and indirect effects in the relationship between teachers’ EI and withdrawal intention showed that work engagement significantly mediated the relationship between EI and withdrawal intention (z = −6.97, *p* < 0.001). The results showed that EI was negatively associated with withdrawal intention (β = -0.164, *p* < 0.001), which in turn affected work engagement (β = 0.429, *p* < 0.001). Although the effect of EI on withdrawal intention was significant (β = -0.141, *p* < 0.001), it became non-significant when work engagement was included as a mediating variable (β = -0.023, *p* = 0.546). This finding supported condition 4 such that the relationship between the independent (i.e., EI) and dependent (i.e., withdrawal intention) variables becomes less intense or non-significant with the mediating variable (i.e., work engagement) included. In sum, the results showed that work engagement fully mediated the relationship between EI and withdrawal intentions, thereby supporting H2.

### 3.4. Tests of Moderated Mediation

**Figure 2:**
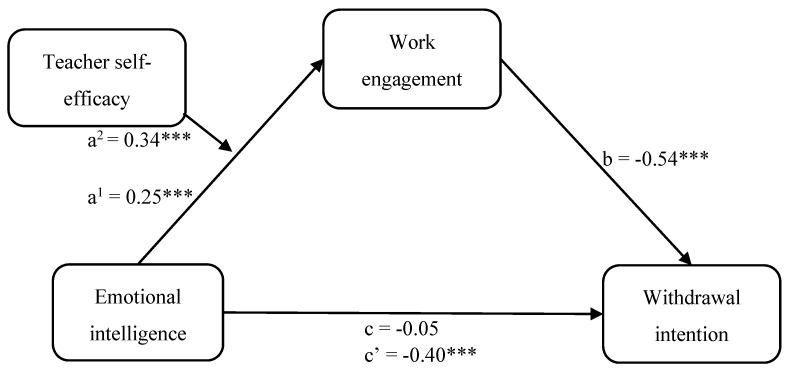
Path analysis of the hypothesized model of teacher self-efficacy as moderator of the relationship among emotional intelligence, work engagement and withdrawal intention. *** *p* < 0.001.

With regard to H3 on the moderating effect of teacher self-efficacy in the mediator model, SEM analyses were conducted to determine whether teacher self-efficacy moderated the relationship between EI and work engagement considering the overall model with withdrawal intentions as an outcome variable. The participants’ age, gender, teaching level, and teaching experience were included in the model as covariates. The path coefficients are shown in Figure 2. The results of the structural equation modeling analyses indicated an excellent fit (*χ^2^* = 26.30, df = 6, CFI = 0.991, TLI = 0.956, RMSEA = 0.070, SRMR = 0.045). In line with standard procedures, the z-statistics were examined regarding the conditional indirect effect [74]. The conditional interaction effect between EI and teacher self-efficacy for predicting work engagement was significant (z = 3.116, *p* = 0.002). Therefore, the results demonstrated a significant moderator effect of teacher self-efficacy on the association between EI and work engagement, thus supporting H3.

**Figure 3:**
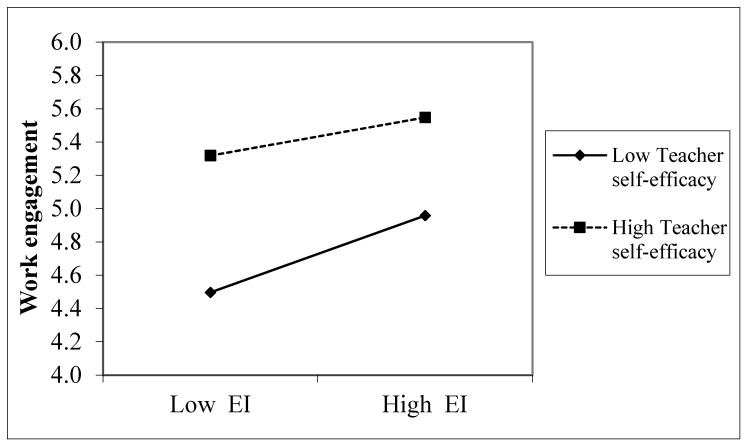
Interaction between EI and teacher self-efficacy on work engagement.

To illustrate the EI × teacher self-efficacy interaction for work engagement, standard guidelines were followed [68]. As shown in Figure 3, the highest mean scores in work engagement were found among teachers reporting high self-efficacy (vs. low self-efficacy). Contrary to expectations, the relationship between EI and work engagement weakened as teacher self-efficacy increased. At low (-1SD) scores in teacher self-efficacy, the relationship between EI and work engagement was positive (β = 0.338, *t* = 6.14, *p* < 0.001), but this relationship decreased for high (+1SD) scores in teacher self-efficacy (β = 0.168, *t* = 2.79, *p* = 0.005).

Following the guidelines of Hayes [75], Johnson-Neyman’s technique was used to determine the region of significance of the values of teacher self-efficacy moderating the relationship between EI and work engagement. This technique analyzes the confidence bands for the values of teacher self-efficacy as a moderating variable in the relationship between EI and work engagement, by means of continuous plots of 95% confidence intervals around simple slopes. Specifically, the range for teacher self-efficacy significant moderation effects were from a minimum mean score of 2.91 (β = 0.604, *t* = 4.41, *p* < 0.001) up to a maximum value of 8.56 (β = 0.134, *t* = 1.96, *p* < 0.05). In sum, although these results support H3 on the moderating effect of teacher self-efficacy in the relationship between EI and work engagement, the findings do not support the expected pattern of a boosting effect for teacher self-efficacy.

## 4. Discussion

The main purpose of this research was to examine the fit of a model in which work engagement mediates the relationship between EI and teachers’ withdrawal intention, examining the moderator role of teacher self-efficacy in the relationship between EI and work engagement. Regarding H1, our results showed that EI was negatively associated with withdrawal intentions among teachers, which is in line with prior research [51] and accords with a recent meta-analytic review supporting the notion that emotionally savvy individuals are less likely to engage in turnover behaviors [28]. Given the implications of teacher attrition for organizations and student achievement, school counselors may want to incorporate EI training programs designed for teacher education and professional development [18]. With regard to H2, the results showed that work engagement totally mediated the association between EI and withdrawal intention. This finding accords with earlier research showing an indirect effect of EI on withdrawal intention [51]. In line with prior research, EI appears to help teachers maintain the effects of pleasant feelings leading to energy and dedication at work [25,49]. Likewise, EI might directly affect occupational well-being through perceived control of environmental factors and display of more effective connections with their work, which might eventually affect teachers’ attitudes toward their occupation and intention to quit [28,37].

Regarding H3, our findings showed that teacher self-efficacy moderated the association between EI and work engagement. Specifically, the results showed that teachers with high EI and high self-efficacy reported higher work engagement than their counterparts perceiving themselves to have fewer personal resources. This finding supports the predictive role of EI on positive emotions among teachers with implications on attitudinal variables such as commitment [76]. In contrast, the lowest scores in work engagement were reported by teachers with low self-efficacy and low EI. This combination of low resources might relate to the way workers deal with their tasks and other environmental factors. For instance, it is likely that teachers with low EI find it harder to perceive supportive relationships at work, which might diminish their levels of motivation [21]. Another underlying mechanism could be that teachers with low EI and low self-efficacy experience fewer mastery episodes within their jobs, which results in lower engagement [18,39]. In sum, these novel findings showed interactive effects of EI and self-efficacy on teachers’ work engagement.

It is worth noting that the results exhibited a pattern contrary to the expectation of a boosting effect of teacher self-efficacy in the relationship between EI and work engagement. The findings showed that EI exhibited a weaker association with work engagement among those teachers with higher self-efficacy. In other words, the results suggested that having high EI was most impactful for teachers reporting low self-efficacy compared to those with high self-efficacy in experiencing higher work engagement. One plausible explanation for this finding could be provided with regard to the compensatory model of EI [77]. Accordingly, in comparison to teachers who perceive themselves as less self-efficacious, teachers with higher self-efficacy might not benefit from having EI to achieve higher work engagement, possibly because teachers who possess one resource such as EI would already attain high work engagement [48]. This compensatory effect was found in previous studies testing the interplay between emotional abilities and cognitive intelligence [77] or workplace social support [78]. 

### 4.1. Theoretical and Practical Implications

There are theoretical and practical contributions for the teacher education literature derived from these results. First, these findings respond to the call for studies focusing on the interplay between individual characteristics and EI to predict workplace outcomes [32]. These novel results, if replicated with larger samples and complementary study designs, would allow researchers to develop more integrative models built on the joint contribution of teachers’ personal resources to work engagement and organizational outcomes such as withdrawal intention [16,54]. These models could drive assessments of personal resources that allow teachers to feel more engaged at work and to intend to stay—or, conversely, assessments of the risk profiles in terms of low self-efficacy and low EI that might predict low engagement and associated withdrawal intentions.

Second, this study could contribute in a practical way, as it adds novel evidence for developing EI training with teaching professionals [79]. Considering these findings, together with earlier research showing baseline levels of EI and self-efficacy as relevant predictors in EI improvements through training [80], teacher education practices could benefit from knowledge of the individual factors (e.g., self-efficacy levels) influencing such EI training.

Following a positive primary preventive approach [14], the current results on the compensatory effects of EI may have implications for further works with early-career teachers reporting low self-efficacy. For instance, intervening at the individual level to develop teachers’ emotional abilities might prove more effective among teachers who are less confident in their skills to engage students, to manage disruptive behaviors in class, or to display instructional strategies in their daily work. In particular, these interventions could be targeted at early-career teachers with few years of teaching experience as this group tend to show less confidence in their teaching skills than their veteran counterparts [13,81]. Since EI has been shown to play a role in teacher motivation and withdrawal, such training should be promoted by teacher preparation programs including group learning or social dynamics [79]. Instructing new teachers on how to develop their emotional abilities would be helpful not only for their satisfaction and effectiveness but also for creating a more supportive environment in which teachers can feel more resourceful to manage everyday demands [16,18]. This may have direct effects not only on their performance but also on their commitment and desire to grow in their career [16]. In view of current attrition rates and trends found among newly qualified teachers [13,81], these practices deserve more efforts to boost teachers’ work engagement and retention.

### 4.2. Limitations and Ideas for Future Research

This study has several limitations suggesting plausible avenues of research. First, data were collected cross-sectionally, thereby precluding causal inferences. Although hypotheses on the relations among the variables were based upon frameworks such as JD-R [20] and EI models [21], further studies should confirm our findings using longitudinal designs. A profitable direction would consist of research addressing longitudinal designs, examining how teacher self-efficacy might moderate the causal link among EI, work engagement, turnover intentions, and other performance outcomes across different time points [43]. 

Second, the namely health-impairment process should be tested in future research to provide evidence regarding the protective role of personal resources (e.g., EI and teacher self-efficacy) on the relationships between job-related demands (e.g., conflicting relationships or violence against teachers), strain, engagement, and withdrawal intentions [82,83]. Following the current knowledge on emotion management within the teaching context [16], researchers could examine which teachers’ emotion-regulation strategies are more effective for reducing the deleterious impact of job demands on withdrawal intentions [84,85]. This research should be conducted with beginning teachers considering patterns of emotional exhaustion and withdrawal within the first years of professional experience [81]. Likewise, a research avenue that would beneficially contribute to this field would be to delve deeper into the boosting effect of job demands on the relationship between EI and teachers’ work engagement [86]. 

Third, the use of self-report measures might lead to inflated results associated with common-method bias. Further studies using performance-based EI tests would contribute to a better understanding of the link between emotional abilities and work engagement [32,49]. Future research is advised to include objective measures (e.g., peer ratings of personal resources) [87]. Relatedly, they should consider diary study designs, which would allow scholars to test the enactment of self- and other-focused regulation abilities in emotionally demanding situations so that teachers experience greater work engagement [20,36]. Nonetheless, the complexity of teacher attrition entails the need to adopt complementary methods to achieve significant theoretical advances in this field. As such, a mixed method approach could provide insightful knowledge on how teachers cope with work-related demands to stay engaged and committed [16]. In sum, these findings may contribute to future research developing and testing more comprehensive models with contextual (e.g., professional environment, and working conditions) and personal (e.g., sociodemographic factors, personality traits, professional skills, and psychological resources) factors as antecedents of teachers’ intentions to leave teaching, and also provide valuable knowledge for future effective training programs to increase teacher retention. 

## 5. Conclusions

This study provided novel findings suggesting the critical role of EI and self-efficacy in the prediction of work engagement and associated teachers’ withdrawal intentions. These results are in line with prior research showing individual differences between leavers and stayers regarding resilient factors to educator stress and burnout, and they extend current research on EI and withdrawal intentions in the teaching context [16]. The results of this study could serve as a starting point for considering boundary conditions in EI interventions such as baseline levels of self-efficacy. Finally, this research could contribute to teaching and teacher education practices so that personal resources are trained and, thus, teachers exhibit higher dedication and a greater commitment to their work.

## Figures and Tables

**Table 1:** Factorial validity of the measures.

	Factorial Structure	χ²	Df	p	χ²/df	CFI	TLI	RMSEA	SRMR	ω	λ	α
Emotional intelligence	16 items, 4 factors, first-order model	326.452	98	0.001	3.331	0.95	0.94	0.05	0.03	0.90	0.91	0.90
Teacher self-efficacy	12 items, 3 factors, first-order model	248.95	36	0.001	6.91	0.96	0.92	0.09	0.03	0.93	0.93	0.93
Work engagement	15 items, 3 factors	380.94	78	0.001	4.88	0.96	0.95	0.07	0.03	0.94	0.95	0.94
Withdrawal intention	3 items, 1 factor	2.185	1	0.139	2.18	0.99	0.99	0.04	0.01	0.94	0.92	0.93

Note: *n* = 702. Df denotes degrees of freedom; CFI is Comparative Fit Index; TLI denotes Tuckers-Lewis Index; RMSEA is Root Mean Square Error of Approximation; SRMR represents Standardized Root Mean Square Residual.

**Table 2:** Descriptive statistics and intercorrelations among variables.

	1	2	3	Mean	SD
1. Emotional intelligence	-			5.52	0.68
2. Teacher self-efficacy	0.53 ^**^	-		7.13	1.02
3. Work engagement	0.43 ^**^	0.53 ^**^	-	5.05	0.87
4. Withdrawal intention	−0.17 ^**^	−0.26 ^**^	−0.34 ^**^	1.81	1.69

** *p* < 0.01.
